# Optimization and Development of Albumin–Biopolymer Bioconjugates with Solubility-Improving Properties

**DOI:** 10.3390/biomedicines9070737

**Published:** 2021-06-26

**Authors:** Zsófia Edit Pápay, Sabrina Magramane, Márton Király, Petra Szalkai, Krisztina Ludányi, Péter Horváth, István Antal

**Affiliations:** 1 Department of Pharmaceutics, Semmelweis University, H-1092 Budapest, Hungary; papay.zsofia@pharma.semmelweis-univ.hu (Z.E.P.); sabrina.magramane.lfgeb@gmail.com (S.M.); kiraly.marton@pharma.semmelweis-univ.hu (M.K.); szalkai.petra@pharma.semmelweis-univ.hu (P.S.); ludanyi.krisztina@pharma.semmelweis-univ.hu (K.L.); 2 Department of Pharmaceutical Chemistry, Semmelweis University, H-1092 Budapest, Hungary; horvath.peter@pharma.semmelweis-univ.hu

**Keywords:** Maillard conjugation, glycoconjugate, bovine serum albumin, albumin bioconjugate, biopolymer, galactomannan, alginate, apigenin

## Abstract

Bioconjugation is an emerging field in the food and pharmaceutical industry. Due to its biocompatibility and high ligand binding capacity, albumin is widely used in modern drug delivery systems. However, the protein is sensitive to environmental stresses; albumin conjugates, on the other hand, have improved functional properties. Biopolymers are gaining interest due to their biodegradability and safety, compared to synthetic polymers. In this study, albumin–biopolymer bioconjugates were prepared by nonenzymatic Maillard reaction at 60 °C and 80% relative humidity. This nonenzymatic conjugation takes place between reducing sugars and available amino groups of a protein in certain conditions. The optimal molar ratio and time for the conjugation were studied by several investigation methods, including circular dichroism and fluorescence spectroscopy, sodium dodecyl sulfate–polyacrylamide gel electrophoresis (SDS–PAGE), and determination of available amino groups with ortho-phthaldialdehyde (OPA) assay. All of the measurements provided evidence for the covalent bonding of albumin and biopolymers, resulting in bioconjugates. Based on the results, a higher molar ratio and longer time are necessary to complete the reaction with the available amino groups. However, the optimal parameters are specific to each given biopolymer. The rheological behavior of the conjugates is characteristic of the initial biopolymer, which can be useful in drug development. Moreover, both the physical characteristics of albumin and the solubility-improving capacity were enhanced. Therefore, the potential use of albumin–biopolymer bioconjugates in the pharmaceutical industry could be considered.

## 1. Introduction

Albumin is a predominant protein found in the blood plasma, representing 60% of the total serum protein. In the body, it functions as a means of transportation and disposal of drugs that bind to it. Therefore, albumin’s extreme binding capability can help increase the distribution of drugs, which is why it is extensively used in drug delivery development [1]. In studies, both bovine serum albumin (BSA) and human serum albumin (HSA) are used. The use of BSA is the most common due to its increased stability, affordability, but mostly, its high structural similarity to HSA. HSA and BSA are particularly similar in structure, with a 76% sequence homology, in addition to a disulfide repetitive arrangement that is rigorously maintained in the structure of both serums. The main difference in their structure is the presence of one tryptophan residue in HSA located at position 214, as opposed to two tryptophan residues in BSA, with the extra one located at position 135, in a hydrophobic cleft [2]. Albumin protein is biodegradable, water soluble, and easy to handle. Due to its structure, it can easily be modified to improve its properties as a drug delivery system [1].

Recently, researchers discovered the functional properties of albumin via Maillard reaction, conjugating it with a carbohydrate (monosaccharide or polysaccharide) to form a glycoconjugate using a safe method. Protein glycosylation is an already known procedure that improves protein stability, as well as the pharmacodynamic and pharmacokinetic properties. The glycosylation process involves the covalent attachment of carbohydrate-based molecules (monosaccharides, polysaccharides, etc.) to the protein surface through an enzymatic reaction [3]. It occurs on the side chains of amino acid residues, most commonly N- and O-linked types of glycosylation. Glycoengineering is usually a cell-based or chemistry-based procedure involving glycosyltransferases [4]. On the contrary, the Maillard reaction, named after L. C. Maillard, is known as nonenzymatic glycosylation, and it occurs in an aqueous or dry medium. This naturally occurring “green” reaction is simple and spontaneous. It is an extremely complex process that takes place between reducing sugars (or reducing sugar ends) and the primer amino groups of proteins by the impact of heat. The reaction can also occur at room temperature but a much slower rate. Recently, it was found that in a natural deep eutectic solvent (NADES) reaction medium, the glycation of BSA could be promoted and the reaction time would then be reduced [5]. In drug delivery, the Maillard reaction can be used as an effective method to prepare conjugated proteins, which can improve protein properties, such as their emulsifying ability, foaming capacity, and stability [6,7,8,9], or enhance biological activities [10] by using conventional or microwave heating [11]. Interestingly, it was reported that BSA and xylose could form Maillard protein gels, which have viscoelastic properties [12].

Nowadays, researchers have placed a strong emphasis on natural polymer-based approaches to develop controlled drug delivery systems and tissue engineering [13]. The use of synthetic polymers for drug delivery purposes is of limited application due to problems in biodegradability and the use of organic solvents, resulting in environmental pollution and other issues. However, natural polymers are considered to be much safer since organic solvents are not required for their processing, and because they are biodegradable in nature, with low immunogenicity. Moreover, biopolymeric nanoparticles have the ability to transport bioactive compounds to tissues or cells. However, their drug binding ability is low [14]. Among them is locust bean gum (LBG), a nonionic, natural mucoadhesive substance. LBG is a high molecular weight branched polysaccharide extracted from the seeds of the carob tree (*Ceratonia siliqua*). It is a nonstarch polysaccharide consisting of galactose and mannose in a 1:4 ratio, hence known as galactomannan. It consists of a (1,4) linked D-mannopyranose backbone with branch points at every fourth or fifth chain unit from their C_6_ positions linked to D-galactose. It means that there are mannose elements from a linear chain linked with branched galactopyranosyl residues at a varying distance of the parent chain, depending on the plant origin. The molecular weight of LBG ranges between 300,000 and 1200,000 Da. LBG needs heating to dissolve completely in water. Being nonionic, its aqueous solubility and viscosity are not affected by the pH or the ionic strength of the liquid medium. LBG has a wide variety of applications in the pharmaceutical and food industries and is approved by the US Food and Drug Administration (FDA) [13,15].

Alginate (ALG) is a naturally occurring anionic polysaccharide, typically extracted from brown algae. In order to obtain alginate, either sodium or calcium chloride should be added. Sodium alginate is a widely used pharmaceutical excipient included in both Ph. Eur and USP NF [16]. ALG has many biomedical applications due to its low toxicity, biocompatibility, and relatively low cost. At the low pH of the stomach, alginates may form a low-density viscous gel barrier to reduce reflux symptoms while floating as a “raft” [17]. It is known to be a linear copolymer containing blocks of (1,4)-linked β-D-mannuronate and α-L-guluronate residues. Alginates extracted from different sources differ in block contents. Therefore, the composition, sequence, block length, and molecular weight are factors affecting the physical properties of alginate and its resultant hydrogels. The molecular weights of commercially available sodium alginates range between 32,000 and 400,000 g/mol with low, medium, and high viscosities. For protein delivery, the medium viscosity is optimal [18].

Glycosylated proteins have been proved to possess improved drug delivery properties. Therefore, a model active substance, apigenin, was selected due to its very low water solubility. Apigenin (4′,5,7-trihydroxyflavone) is a natural flavonoid found in abundance in several plants, fruits, and vegetables such as chamomile, parsley, or celery. It provides a wide range of advantageous health properties such as antioxidant, anti-inflammatory, and anticancer. Apigenin is also useful in combating a variety of diseases [19]. However, apigenin possesses poor biopharmaceutical properties among which a high hydrophobicity, high permeability, and low solubility, which make its direct application rather limited despite its numerous advantages. As mentioned, apigenin has a quite low aqueous solubility (1.35 mg/mL) and high permeability (log *p* value = 2.87) and is therefore classified as a BCS (Biopharmaceutical Classification System) II molecule [20,21]. Its water insolubility and poor absorption prevent its clinical use but lead to promising formulation approaches. Based on native mass spectrometry and molecular modeling studies, the binding of apigenin to serum albumin has been located in the tryptophan residue at position 214 (subdomain IIA, site 1) of HSA, and in the tryptophan residue at position 135 in BSA [22,23].

Bioconjugation of proteins with biopolymers could hold many advantages. However, only a few studies address the interaction between serum albumin and polysaccharides with a reducing end such as locust bean gum and alginate, obtaining protein–biopolymer conjugates via Maillard reaction. One study describes BSA–galactomannan conjugates, in which 1 mol BSA bounded 2.5–7 mol of galactomannan (from guar gum). Moreover, another study describes the conjugation of BSA with alginate in a 2:1 molar ratio [8,24]. Therefore, the aim of this study was to prepare albumin bioconjugates (biopolymer-conjugated albumin) by utilizing the nonenzymatic “green” Maillard reaction. Bovine serum albumin, as protein, and locust bean gum (branched structure) or alginate (linear structure), as biodegradable biopolymers, were used. The molar ratio of protein and biopolymers were investigated, as well as the time dependence of the reaction. The effect of conjugates on the solubility improvement of apigenin was also tested.

## 2. Materials and Methods

### 2.1. Materials

Bovine serum albumin was purchased to engineer the albumin conjugates. Bovine serum albumin (BSA), locust bean gum (LBG, M_w_ = 66,000 g/mol), and sodium alginate (ALG, medium viscosity, M_w_ = 120,000 g/mol) were purchased from Sigma Aldrich Ltd. (Hamburg, Germany). The Fluoraldehyde™ o-Phthaldialdehyde Reagent Solution (OPA) was obtained from Thermo Scientific™ (Waltham, MA, USA). Broad-range SDS–PAGE molecular weight standards purchased from VWR (VWR International Ltd., Debrecen, Hungary) were used as molecular mass standards (myosin 200 kDa, β-galactosidase 120 kDa, bovine serum albumin 91 kDa, glutamic dehydrogenase 62 kDa, ovalbumin 47 kDa, carbonic anhydrase 37 kDa, myoglobin 28 kDa, lysozyme 19 kDa, aprotinin 9 kDa). The distilled water was prepared by a laboratory purification system.

### 2.2. Methods

#### 2.2.1. Preparation of Albumin Conjugates with Biopolymers

The BSA–LBG conjugates in 1:3 and 1:6 molar ratios and BSA–ALG in 1:1 and 1:3 molar ratios were prepared by the Maillard reaction in a dry state.

To summarize, the biopolymers (LBG or ALG) were first prepared in distilled water. Afterward, BSA was dissolved in the gel and then lyophilized in order to evaporate the water. Freeze drying of the samples was performed in Scanvac CoolSafe 100 9 Pro type equipment (LaboGene ApS, Lynge, Denmark). The freeze-drying process was controlled by a computer program (Scanlaf CTS16a02), and all of the parameters were recorded continuously. The process parameters (Table S1) are shown in the Supplemetary Materials.

For the next step, the samples were kept under controlled temperature (60 °C) and 80% relative humidity (with saturated KCl solution) for hours to days (Table 1). For further analysis, the samples were kept in a desiccator or redissolved in water. For purification, the redissolved products were filtered through a 0.45 µm pore-sized cellulose acetate membrane filter (Q-Max^®^ Frisenette ApS, Knebe, Denmark).

#### 2.2.2. FTIR and DSC Measurements

Fourier transform infrared spectroscopy (FTIR) spectra of the conjugated albumin samples were evaluated using a Jasco FT/IR 4200 spectrometer equipped with Jasco ATR PRO470 H single reflection accessory (ABL&E-JASCO Ltd., Budapest, Hungary). The measurements were performed in absorbance mode. Approximately 3 mg of the solid samples were placed between the plate and the probe. The spectra were recorded with 50 scans, in a frequency range between 1000 to 4000 cm^−1^ and a resolution of 4 cm^−1^ at room temperature. The data were analyzed using the FTIR software (Spectra Manager II, ABL&E-JASCO Ltd., Budapest, Hungary).

The conjugates were also characterized by differential scanning calorimetry, using Exstar 6000 DSC (Seiko Instruments Inc., Chiba, Japan). Samples (3 and 5 mg) were weighed accurately and analyzed in aluminum pans. The pans were equilibrated to 10 °C, and the thermal analyses were carried out from 10 to 340 °C with 2 °C/min speed. The data were analyzed with Spectra Analysis software (Seiko Instruments Inc., Chiba, Japan).

#### 2.2.3. Rheological Measurements

The rheological properties of BSA conjugates before and after the Maillard reaction were measured with a Kinexus Pro+ rheometer using parallel plate geometry (CP1/50 SR1222 SS, Solvent Trap 55 mm C0157 SS) (Malvern Instruments Ltd., Malvern, UK). The measured data were registered and analyzed with rSpace for Kinexus Pro+ 1.3 software. The gap between the two sample placement plates was 55 mm. The number of samples per decade value was 10. The temperature of the samples was set to 25.0 °C and controlled with an accuracy of ± 0.1 °C by the rheometer.

The oscillatory measurements were performed at a shear strain amplitude of 60% within the linear viscoelastic region. These data were based on preliminary amplitude sweep test results. The frequency was in the range of 0.1–100 Hz. Elastic moduli (G’), viscous moduli (G”), and the phase shift angle (δ) were determined using an oscillatory rheometer. For the analysis of the viscoelasticity, their points of intersection were studied. For all the measurements, a stainless steel cylindrical cover was placed over the samples in order to prevent evaporation. Three parallels were recorded, and the mean values were displayed.

#### 2.2.4. Fluorescence Spectroscopy Measurements

The fluorescence emission spectra of BSA and BSA conjugates were measured with the Jobin Yvon Horiba Fluoromax 3 (Jobin Yvon Horiba Inc., Paris, France) spectrofluorometer. The samples were diluted 10 times, and the fluorescence emission spectra were recorded between 300 and 450 nm at 25 °C where the excitation wavelength was set to 285 nm. The data collection frequency was 0.5 nm, and the integration time was 0.2 s. The emission slit was set at a bandpass width of 5 nm, and the excitation slit at 5 nm. Each spectrum was recorded three times, and the mean values were calculated automatically. SPSERV V3.14 software (^®^ Csaba Bagyinka, Institute of Biophysics, Biological Research Center of the Hungarian Academy of Sciences, Szeged, Hungary) was used for baseline correction, for five-point linear smoothing, and the correction to the wavelength-dependent sensitivity changes of the spectrofluorometer. The subtraction of the Raman band at 390 nm was performed.

#### 2.2.5. Determination of Available Amino Groups

In order to assess the extent of glycation, ortho-phthaldialdehyde (OPA) assay was applied. The primer amine detection with Fluoraldehyde (OPA) Reagent Solution was conducted by following standard protocol; briefly, the Fluoraldehyde Reagent Solution and samples at room temperature were equilibrated before use. As the first step, a set of BSA standards of known concentrations were prepared by dissolving the BSA in the same diluent as the unknown sample (distilled water). Then, 4000 µL reagent was added to 400 µL of each sample (BSA or modified BSA solutions). The sample diluent (distilled water) was used as a blank. After vigorous stirring and a two-minute waiting period, the absorbance was measured at a wavelength of 340 nm. In order to obtain the best possible results, all samples were measured at the same time interval after mixing. The absorbance of the standards versus concentration was plotted. By using the standard equation, the concentrations of the BSA conjugates were determined. The measurement was conducted in triplicate.

#### 2.2.6. Electrophoretic Analysis

Sodium dodecyl sulfate–polyacrylamide (SDS–PAGE) gel electrophoresis was carried out using a 10% Tris-Glycin gel and a vertical Novex Minigel electrophoretic system. A total of 100 μL of each sample was mixed to 100 μL SDS sample buffer (62.5 mM Tris–HCl, 2% SDS, 25% glycerol, 0.01% bromophenol blue, 5% mercapto-ethanol or 100 mM DTT, pH = 6.8) and denatured at 80 °C for 5 min. Next, 15 μL of each sample was used, and the electrophoresis was carried out at 35 mA and 150 V for 90 min until the bromophenol blue reached the lower edges of the gel. Protein bands were visualized by staining with Coomassie Brilliant Blue R-250 in 50% methanol and 10% acetic acid staining solution, distained by a solution of 5% methanol and 10% acetic acid overnight. All samples, including the standard sample, contained the same amount of initial BSA in order to be comparable. In the case of alginate samples, this was 1 mg/mL, and for the samples containing LBG, it was 0.5 mg/mL.

#### 2.2.7. Circular Dichroism (CD) Spectroscopy

The circular dichroism (CD) and UV spectra were recorded on a Jasco J-720 spectropolarimeter (Jasco, Inc., Easton, Maryland, USA) with different parameters. In the case of protein structure change tracking, the wavelength was set in the range of 190–280 nm. The measurements were performed in 0.1 and 0.2 cm cylindrical cuvettes with 50 nm registration speed, 3× accumulation, 0.1 nm data interval, 100 mdeg sensitivity, and at 1 nm slit. Distilled water was used as a control. Data were expressed as the ellipticity (θ, mdeg), and g-values were calculated based on the following equation:

(1) $$g=\frac{\theta }{A}=\frac{3298.2\times ∆\varepsilon \times c\times l}{\varepsilon \times c\times l}$$

where *θ* is the measured ellipticity, which can be expressed with the ∆*ε* value. ∆*ε* is the difference of the molar absorption of the right- and left circularly polarized light components. *ε* is the molar absorption coefficient. The concentration ‘*c*’ and path length ‘*l*’ can be found in both the nominator and denominator; therefore, the equation can be simplified.

#### 2.2.8. Solubility Improvement of Apigenin

Apigenin stock solution (0.8 mg/mL) was prepared in methanol to ensure the same apigenin concentration in each sample. After that, 1 mL of stock solution was precisely measured into test tubes, and the methanol was evaporated using a laboratory nitrogen evaporator. Then, 4 mL solution of BSA–LBG 1:6 (5 days) or BSA–ALG 1:3 (48 h) were added and vigorously stirred. The apigenin solution in water (approximately 1 µg/mL) and BSA stock solution (with the same concentration as the bioconjugates) were used as control. The CD and UV spectra were recorded on a Jasco J-720 spectropolarimeter (Jasco, Inc., Easton, MD, USA) with different parameters. When the complex formation was followed, a 1.0 cm rectangular cuvette was used in the 250–450 nm wavelength range with 100 nm registration speed, 5× accumulation, 0.1 nm data interval, 10 mdeg sensitivity, and at 1 nm slit.

## 3. Results

### 3.1. FTIR Measurements

Fourier transform infrared spectroscopy (FTIR) is a widely used technique to investigate the interactions between proteins and carbohydrates. It makes it possible to detect the differences in the functional groups of the conjugates through the peak intensity, allowing quick and efficient identification of the compounds according to their functional groups and bond vibrations.

**Figure 1:**
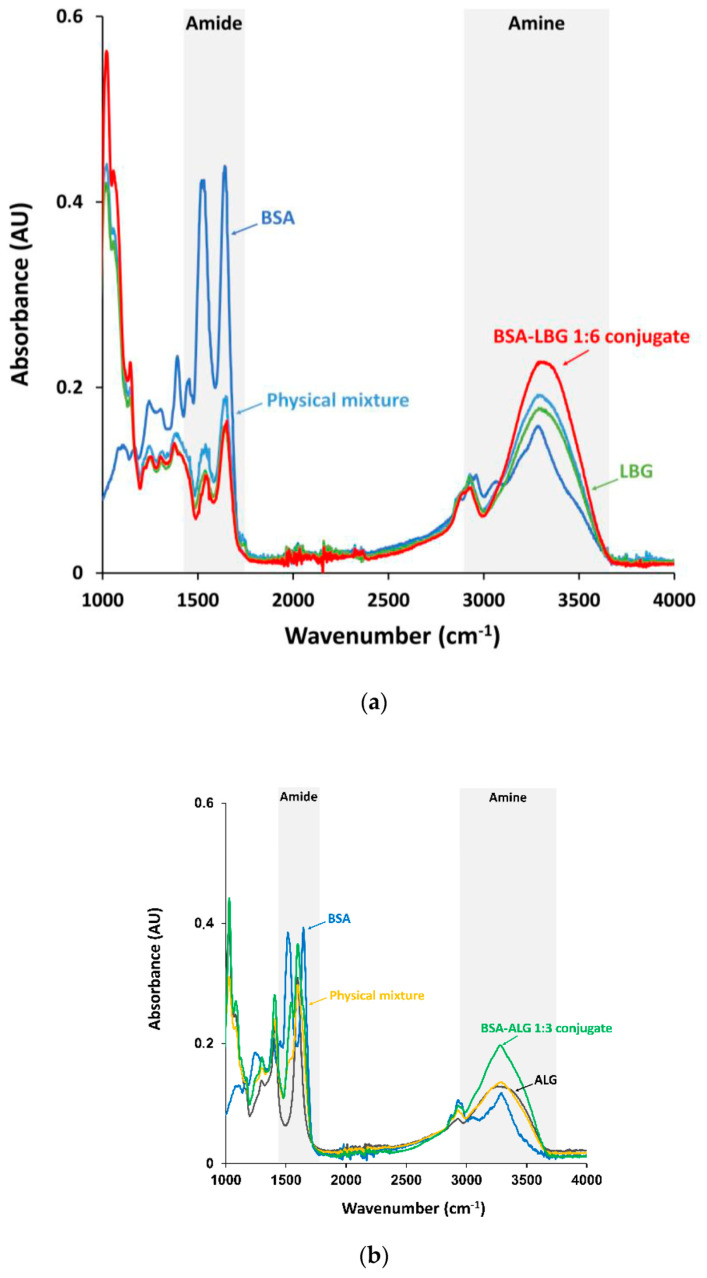
(**a**) FTIR absorption spectra of the native BSA, LBG, BSA–LBG physical mixture and BSA–LBG 1:6 conjugate; (**b**) FTIR absorption spectra of the native BSA, ALG, BSA–ALG physical mixture, and BSA–ALG 1:3 conjugate.

Figure 1a shows the absorption spectra of native BSA and BSA–LBG conjugates. In the spectrum of BSA protein, the amide I band at 1635 cm^−1^ (mainly C = O stretch) and the amide II band at 1530–1500 cm^−1^ (C-N stretching and N-H bend) can be seen. The medium broad peak at 3276 cm^−1^ corresponds to bonded N-H stretch of amide, and a smaller band at 1057 cm^−1^ is the C-N stretch of aliphatic amine. For pure LBG, the band at 3430 cm^−1^ represents O–H stretching vibration. The band at 2924 cm^−1^ is due to C–H stretching of the –CH2 groups, while the absorption bands at around 2921 and 2877 cm^−1^ can be attributed to C-H symmetric and asymmetric stretching, respectively. Due to ring stretching of galactose and mannose, the bands appear at 1641 and 1657 cm^−1^. In addition, the bands in the region of 1350–1450 cm^−1^ are due to symmetrical deformations of CH2 and COH groups. The bands resulting from the primary alcoholic –CH2OH stretching mode and CH2 twisting vibrations appear at 1078 and 1021 cm^−1^, respectively [9].

Figure 1b indicates the BSA–alginate conjugates, in which pure sodium alginate displayed absorption bands applicable to hydroxyl, ether, and carboxylic functional groups. Stretching vibrations of O–H bonds of alginate appeared in the range of 3000–3600 cm^−1^. Stretching vibrations of aliphatic C–H can be observed at 2920–2850 cm^−1^. Absorption bands at 1649 and 1460 cm^−1^ are due to the asymmetric and symmetric stretching vibrations of carboxylate salt ion, respectively. The bands at 1107 and 935 cm^−1^ could be attributed to the C–O stretching vibration, and the C–O stretching with contributions from C–C–H and C–O–H deformation.

The glycosylamine Maillard products, both LBG and ALG conjugated, showed a significant increase in amine (~3400 cm^−1^) absorption band, compared to the native protein and the physical mixtures. On the contrary, the intensity of amide I and II bands (~1500 cm^−1^) decreased when the protein was conjugated, suggesting conformational changes of the protein.

### 3.2. DSC Measurements

**Figure 2:**
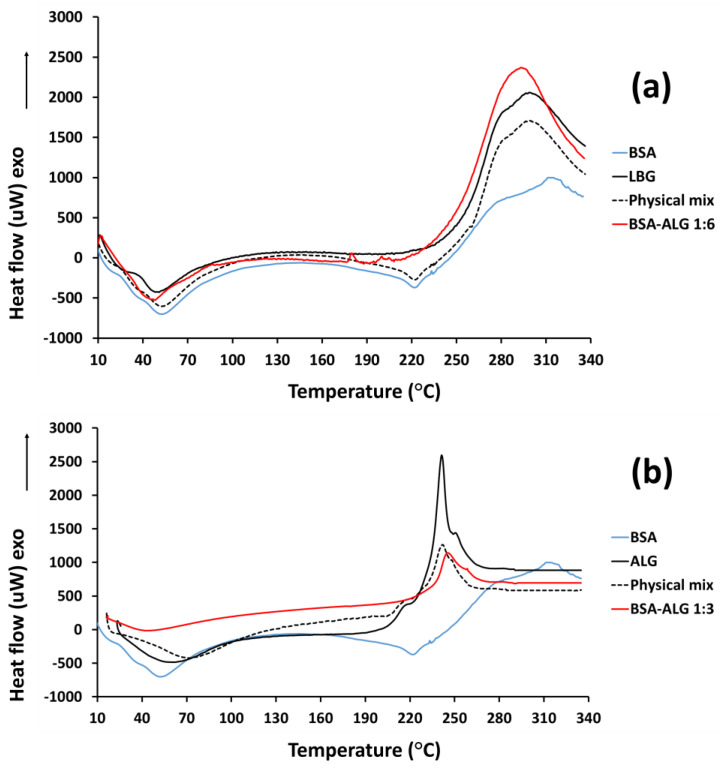
(**a**) DSC thermograms of the native BSA (blue line), LBG (black line), BSA–LBG physical mixture (dotted line), and BSA–LBG 1:6 conjugate (red line); (**b**) DSC thermograms of the native BSA (blue line), ALG (black line), BSA–ALG physical mixture (dotted line), and BSA–ALG 1:3 conjugate (red line).

Figure 2 exhibits the differential scanning calorimetry (DSC) analysis of the samples, indicating changes in the structure of the native BSA. The broad endothermic peak on the thermograms 50–100 °C refers to the evaporation of water in all samples. The thermogram of native albumin showed a melting point around 60 °C and abroad peak at 220 °C due to the structure change and denaturation of the protein. LBG exhibited a broad exothermic peak indicating the glass transition temperature at 270 °C. In the case of ALG, as the temperature increased to 250 °C, decomposition of the polymers occurred, as shown by the upward movement of the thermogram in Figure 2b.

In the case of conjugates, the endothermic peaks of albumin denaturation were not characteristic, differing from the physical mixtures. However, the exothermic peaks suggesting the decomposition of glycosylamines were characteristic of the attached biopolymer. These findings support the increased thermal stability of the bioconjugates, compared to albumin.

### 3.3. Rheological Measurements

**Figure 3:**
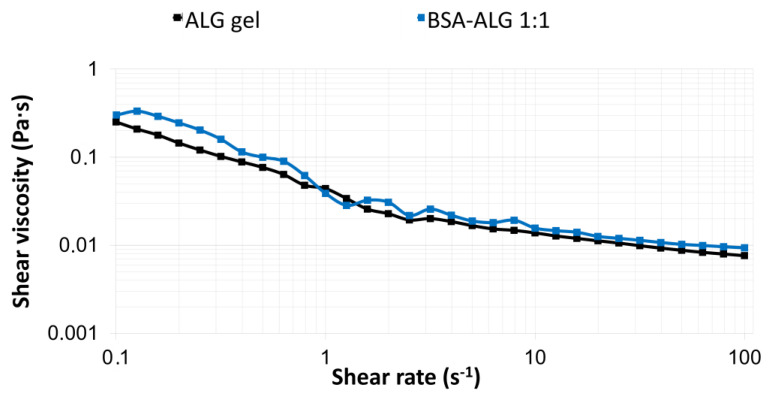
Flow curves contained viscosity (Pa·s) vs. shear rate (s^−^^1^) of alginate samples before (black line) and after conjugation (blue line).

Figure 3 shows the rheological behavior of alginate biopolymer with medium viscosity subjected to steady shear in the range of 0.1–100 1/s. ALG is not viscoelastic in nature; therefore, shear viscosity was measured, and as the viscosity decreased by increasing the shear rate, the shear-thinning behavior was shown. The viscosity of the initial ALG gel was 0.3 Pa·s. In correspondence to the literature [18], it can be seen that the rheological behavior was not altered due to the conjugation (BSA–ALG 1:3, 48 h) when compared to the initial ALG solution.

**Figure 4:**
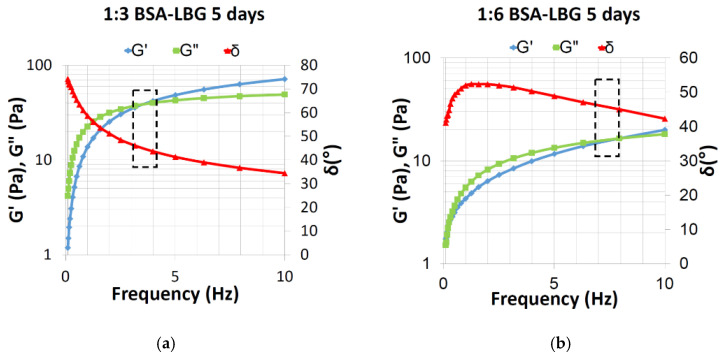
Storage modulus (blue line, G′), loss modulus (green line, G″) and phase angle (red line, δ) for BSA–LBG 1:3 and BSA–LBG 1:6 conjugates (25 °C).

It is known that LBG possesses a viscoelastic behavior; the viscosity of the initial LBG solution was 0.279 Pa·s. To characterize the viscoelastic rheological properties of LBG conjugates, phase angle δ (°), loss modulus (viscous modulus) G” (Pa), and storage modulus (elastic modulus) G’ (Pa) were all measured with the angular frequency (Hz) used, as shown in Figure 4. The value of phase angle (δ) crossed 45° at which point G’ = G”, indicating the viscoelastic rheological behavior of the 1:3 and 1:6 BSA–LBG conjugates.

Table 2 shows the calculated values of G” and G’ at 45° phase angle based on the measured data using a linear interpolation process. The results were in accordance with Figure 4: the cross-section and therefore the viscoelasticity was verified for BSA–LBG bioconjugates.

### 3.4. Fluorescence Spectroscopy Measurements

**Figure 5:**
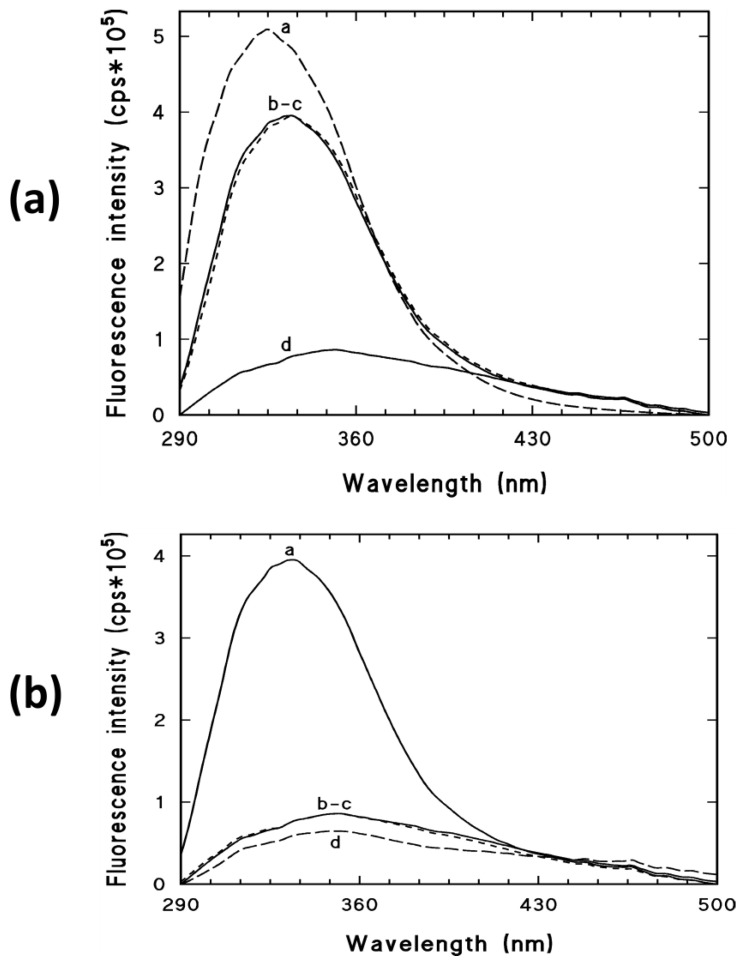
(**a**) The fluorescence emission spectra of BSA solution (a), LBG and physical mixture of BSA–LBG 1:6 (b–c), and BSA–LBG 1:6 conjugate (d); (**b**) the fluorescence emission spectra of BSA solution (a), BSA–LBG 1:3 and 1:6 conjugates (b–c), and when added apigenin (d).

Figure 5a demonstrates the fluorescence emission spectra of the BSA solution, LBG, BSA–LBG 1:6 physical mixture, and BSA–LBG 1:6 conjugate. The fluorescence intensity of the BSA-LBG physical mixture decreased, compared to the BSA solution with no obvious shift of the maximum position, resulting in similar emission spectra to the pure LBG. The significantly lower emission intensity of BSA–LBG 1:6 conjugates indicates a Maillard reaction and probably conformation changes of the protein, which result in a quenching mechanism of the fluorescence emission spectrum of native BSA.

Figure 5b shows that both BSA–LBG 1:3 and 1:6 provided significantly lower emission spectra. The quenching effect was even more prominent when apigenin was added to the solution, suggesting a binding to the tryptophan (Trp) region of BSA. Similar results were observed and the quenching effect occurred in the case of BSA–ALG conjugates as well (data not shown).

### 3.5. Determination of Available Amino Groups

**Figure 6:**
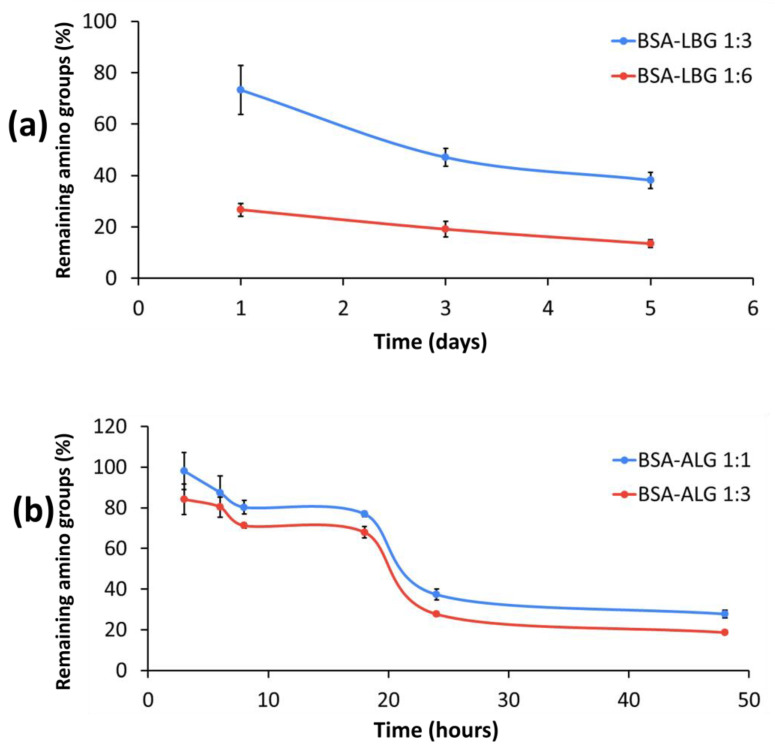
Remaining free primer amino groups of BSA conjugates: (**a**) BSA–ALG 1:1 (blue line) and 1:3 (red line); (**b**) BSA–LBG 1:3 (blue line) and 1:6 (red line).

The OPA reaction measurement, which shows the remaining primer amino groups of BSA, was in good correlation with the gel electrophoresis data. The principle of the reaction is that the unreacted amino acids of BSA can bind to the thiol group of OPA, and the amount can be determined by the spectroscopic method. In both BSA–ALG 1:1 and 1:3 molar ratios, after 18 h, a prominent decrease could be observed, and after 48 h, less than 20% unreacted primer amino groups could be detected. Figure 6 also shows that a higher molar ratio of biopolymers provided more efficient attachment in the primer amino groups of BSA. It can clearly be seen that BSA-LBG 1:6 conjugate provided more effective glycation even after the first day, compared to the 1:3 molar ratio. After 5 days, the free primer amino groups decreased below 20%. We concluded that a higher molar ratio and at least 24 h are necessary for the reaction in the case of both biopolymers.

### 3.6. Electrophoretic Analysis

**Figure 7:**
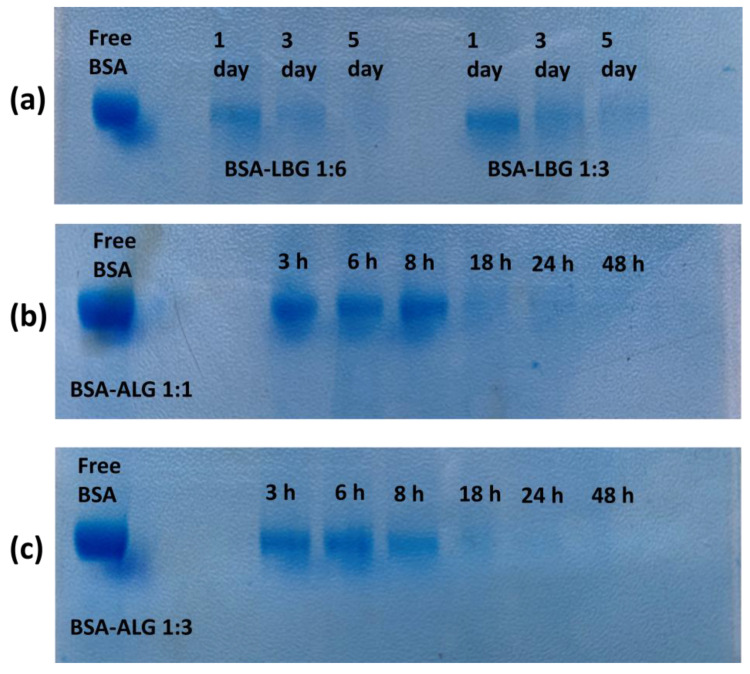
Sodium dodecyl sulfate–poly acrylamide gel electrophoresis pattern of BSA conjugates: (**a**) BSA–LBG 1:3 and 1:6; (**b**) BSA–ALG 1:1; (**c**) BSA–ALG 1:3.

SDS–PAGE gel electrophoresis was conducted to confirm the formation of albumin and biopolymer conjugates by the Maillard reaction. Figure 7 shows the result of the gel electrophoresis of all the conjugates. The blue stains indicate the free BSA on the gel. The less intense the color was, the less free (not conjugated) BSA could be detected. Therefore, based on the intensity of the color, we could determine the remaining free BSA concentration in the samples and the effectiveness of the Maillard reaction.

In the case of BSA–LBG 1:3 and 1:6 samples, either the amount of LBG was not sufficient or the time (5 days) was not enough to complete the Maillard reaction. A small amount of BSA could be observed even after 5 days. Based on the faded stains, the 1:6 molar ratio is more efficient in conjugation (Figure 7a).

In the case of BSA–ALG 1:1 and 1:3 conjugates (Figure 7b,c), the amount of alginate biopolymer was sufficient to react with all the BSA molecules. The more ALG concentration (1:3 molar ratio) resulted in a faded stain, the less BSA remained in the samples. After 24 h, no stain could be detected by the naked eye, which is in accordance with the OPA results and CD spectra. After 48 h, no stain could be detected. Therefore, we concluded that the Maillard reaction was completed.

### 3.7. Circular Dichroism (CD) Spectroscopy

**Figure 8:**
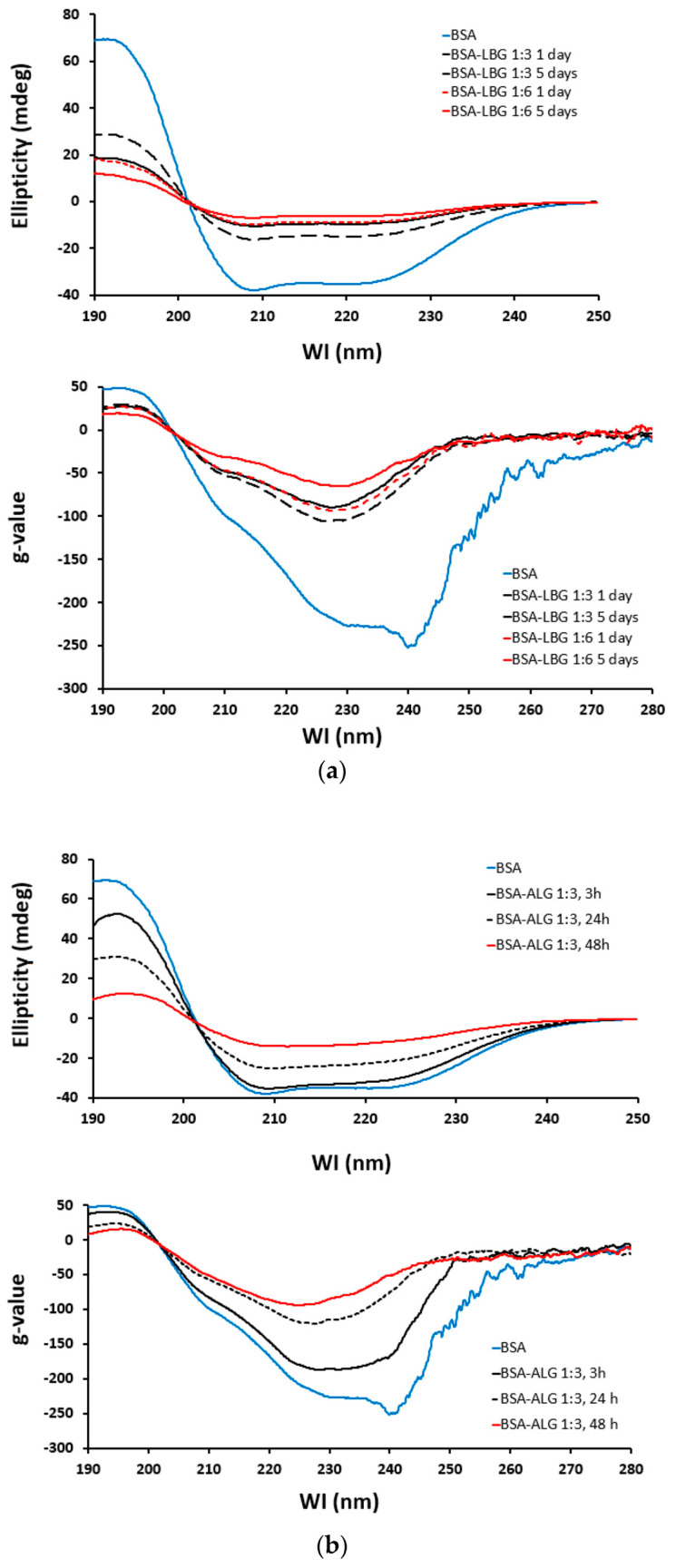
CD (upper) and G spectra (lower) of various BSA conjugates with increasing reaction time: (**a**) BSA (blue), BSA–LBG 1:3 conjugates (black dashed line, 1 day and black solid line, 5 days), and BSA–LBG 1:6 (red dashed line, 1 day and red solid line, 5 days); (**b**) BSA (blue), BSA–ALG 1:3 3 h (black solid line), 24 h (black dashed line), and 48 h (red solid line).

Compared to the native BSA as a standard, the effect of time and molar ratio of conjugates can be observed on the CD spectra and by plotting the g-values (Figure 8). It shows that the “g” spectrum is a concentration-independent inherent parameter for the molecule. If any structural change occurs, which affects either the CD or the UV signal, the result will be a change in the “g” spectrum. According to Figure 8a, the conjugation with LBG proved to be more effective in the 1:6 molar ratio for 5 days. In the case of ALG, the best result could be obtained with the 1:3 molar ratio for 48 h (Figure 8b). However, only a slight change could be observed from 24 h to 48 h. All of these results are in a good correlation with all of the above-measured parameters.

### 3.8. Solubility Improvement of Apigenin

**Figure 9:**
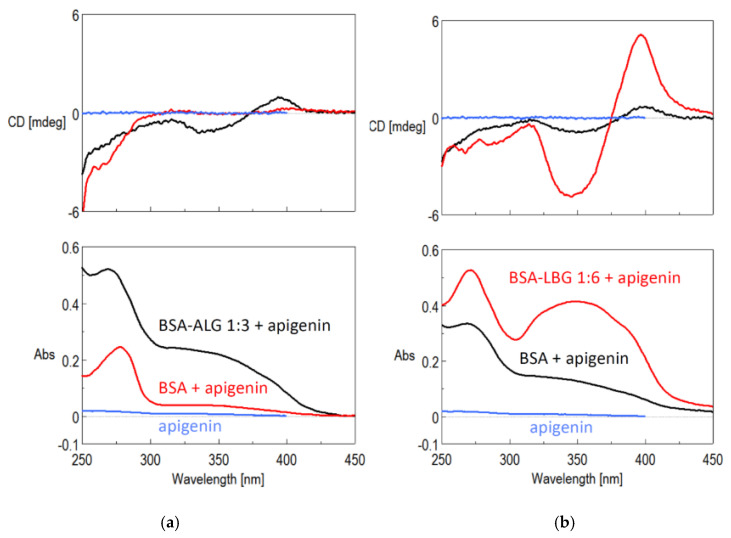
(**a**) ICD (upper) and UV (bottom) spectra of aqueous solution of apigenin (blue), the BSA–apigenin complex (black), and the BSA–ALG 1:3–apigenin complex (red); (**b**) ICD (upper) and UV spectra (lower) of aqueous solution of apigenin (blue), the BSA–apigenin complex (red) and the BSA–LBG 1:6–apigenin complex (black).

A weakly induced circular dichroism (ICD) spectrum indicated the formation of the BSA apigenin complex, while the UV signal of this complex became stronger, compared to the aqueous apigenin signal. The modified BSA showed significantly stronger ICD and UV signals. The CD provided evidence for the complex formation, while the UV spectrum indicated a better dissolution of apigenin in the presence of the BSA–ALG bioconjugates (Figure 9a). Similar trends could be observed in the case of LBG modified BSA. For that, the intensity was much higher, which can either be a result of the solubility increase or the larger BSA and BSA–LBG concentrations (Figure 9b). Therefore, further solubility studies should be conducted.

## 4. Discussion

The Maillard reaction is a promising approach for protein modification. Nonenzymatic glycation of proteins with mono- or polysaccharides leading to Maillard reaction products (MRPs), could improve their functional properties such as solubility, thermal stability, and viscosity. Moreover, improved emulsion formation, foam formation, and encapsulation could be achieved. Lately, several enhanced biological properties of MRPs were published such as antioxidant activity, antimicrobial activity, and antitumor effect [25]. Generally, the Maillard reaction shows the browning of compounds due to the interactions between carbonyl groups such as reducing sugars and amino compounds, namely, amines, peptides, or proteins. It is known that this nonenzymatic reaction usually occurs during the processing of foods (heating and storage); however, it can also occur in the body, resulting in toxic advanced glycation end products (AGEs) [26]. The Maillard reaction starts with a reducing sugar reacting with an amine, creating glycosylamine. These substances (if the reaction condition is maintained) can later undergo a so-called Amadori rearrangement to produce a derivate. The reaction is continuous and very reactive intermediate substances are formed, which subsequently react in several different ways, producing dark-colored insoluble materials, which may cause harmful effects [25,27]. The effect of processing conditions such as reaction temperature, incubation time, and reactant influence the outcome of the reaction [27]. Therefore, the aim of this study was to stop the reaction at the initial stage, when the carbonyl group reacts with a free amino group, resulting in the formation of N-glycosylamines, and to optimize the incubation time and molar ratio of the LBG and ALG biopolymers.

In this study, the successful Maillard reaction between BSA and LBG or ALG polysaccharides was verified by several investigation methods. FTIR analysis has been widely used to study the characteristics of BSA and LBG or ALG biopolymers and to confirm the formation of the conjugates through the Maillard reaction. The intensity of amine IR (infrared) absorptions (~3400 cm^−1^) was increased due to the presence of glycosylamine products, while that of the amide I (~1665 cm^−1^) and II (~1530 cm^−1^) bands were significantly decreased on the spectra of BSA conjugates, which indicates the conformational changes of the protein. This was prominent in the case of BSA–LBG 1:6 and BSA–ALG 1:3 bioconjugates. Similar results were concluded by Barbosa et al., who described the decrease of the amide region of casein due to the covalent bond formation with LBG via Maillard reaction [9].

DSC is a widely used thermal analytical technique that provides insights into the physical state of substances and gives information about the changes in the structure of a protein. Moreover, thermal stability is an important parameter in the pharmaceutical industry. A high temperature may damage proteins, even albumin, which is stable up to 60 °C. However, Maillard conjugation has been successfully used to improve the thermal stability of albumin [25]. The DSC thermograms of the BSA–LBG and BSA–ALG bioconjugates are not characteristic of the native albumin and show better thermal stability with a lower enthalpy change, probably due to the exothermic conjugation reaction.

It has been reported that the viscosity of a formulation plays a significant role in the effectiveness of drug delivery. For example, several body fluids such as synovial fluids, mucins, or tear film possess viscoelastic behavior. This kind of rheological property is particularly important in formulations. The use of viscoelastic formulations offers therapeutic advantages, such as facilitating the retention and permitting the easy spreading of the formulation at the same time. The viscoelastic rheological behavior shows the properties of an elastic solid under low force and then viscous under higher force. This behavior can be investigated by oscillation viscosity measurements (rheometers), in which case the change in the value of the applied shear stress describes a sinusoidal curve. The shear stress causes shear deformation in the sample. If the sample has viscoelastic properties, the deformation will follow the stress with a phase difference. The phase difference is described by the phase shift angle (δ), which in this case is 45°. If the values of the viscous (G”) and elastic (G’) modulus are the same, then δ = 45°.The intersection of G’ and G” assigns the transition point of viscoelastic materials, which characterizes the transition from viscous type behavior into elastic type behavior in case of viscoelastic liquid. This intersection and the phase angle are highlighted in Figure 4. The oscillation rheological measurement proved that the viscoelastic properties of the BSA–LBG conjugates were maintained at both molar ratios.

The rheological behaviors of BSA–ALG conjugates were compared to the initial albumin and alginate mixture. It can be concluded that the rheological properties were not altered after Maillard conjugation and that the shear-thinning behavior was maintained. This viscosity could make the albumin–ALG conjugates a suitable carrier for cells or drug delivery [28], and it was recently revealed that alginate-containing particle elasticity directs tumor accumulation, suggesting that it can design a parameter to enhance tumor delivery efficiency as well [29]. On the contrary, in another study, a significant increase was observed in the viscosity of BSA–alginate solution, compared to a nonheated protein–polysaccharide solution. However, this could be attributed to the different molar ratios of the compounds (BSA–ALG 2:1) [24].

Fluorescence spectroscopic measurements are a useful tool to investigate the conformational changes of albumin because they have an intrinsic fluorescence emission in the Trp region. Fluorometric spectroscopic studies were performed to characterize changes in the tryptophan surroundings with the conjugation. The emission spectra showed that the maximum fluorescence intensity was significantly decreased (by about 80%) due to the conjugation of either LBG or ALG biopolymers.

Fluorescence quenching is a process during which the fluorescence intensity of a component is decreased, which can be accomplished by numerous molecular interactions. The phenomenon of fluorescence quenching can result from various inter and intramolecular interactions such as complex formation or conformational changes. This refers to a stable nonfluorescent complex that is formed by a quencher with the fluorophore. In this case, the quenchers were the biopolymers, and the fluorophore was the albumin. This suggests that the conjugation partially affected the side chains of proteins in tertiary structure without changing the native structure [8,28].

The quenching effect of the Maillard reaction on fluorescence intensity of serum albumins (BSA and HSA) has been studied previously, but there are no data related to its behavior in the case of apigenin. Adding apigenin to the solution resulted in the quenching of the albumin bioconjugates, suggesting that the apigenin molecule can bind to the BSA, which affects the conformation of the Trp microregion. A study conducted by Zhao et al. [30] showed that apigenin engendered strong BSA fluorescence quenching mechanisms, which can be due to a static quenching process and van der Waals forces, with hydrogen bond being a key part in the binding process. Ultimately, it was found that the binding of apigenin to BSA is a consequence of a static mechanism by hydrophobic and electrostatic force and is located on the tryptophan residues in the hydrophobic pocket of BSA. It was concluded that apigenin could bind to the Trp region, and the spectral maximum was affected. Therefore, hydrophobicity and polarity of the fluorophore residues are probably altered, but the secondary structure of serum albumin is not altered in correlation with the CD spectra.

The remaining free amino groups of BSA can be determined with o-phthaldialdehyde (OPA) method because OPA reacts in the presence of thiols, especially with primary amines above their isoelectric point. During conjugation, side amino acid groups of albumins react with carbonyl groups of the reducing end of the biopolymer to form a conjugate. Since there is remaining amino group content, the extent of the Maillard reaction can be evaluated by the OPA reaction. In the conjugate solution, the decrease in absorbance value is in correlation with the decrease in free amino groups’ content. Based on the result, a longer incubation time and a higher molar ratio of the biopolymer resulted in an enhanced reaction rate: in the case of LBG 1:6, for 5 days, and in the case of ALG 1:3, for 48 h (2 days). Similar findings were observed about the effect of incubation time and albumin:reactant ratio in the mixture [8,11]. Our results confirm that the type of polysaccharide is important in the glycation, as well as that of monosaccharides [31].

The success of the reaction was also supported by SDS–PAGE gel electrophoresis with Coomassie Blue and Periodic acid–Schiff (PAS) staining method. Gel electrophoresis evaluated the extent of the conjugation with different molar ratios of LBG and ALG biopolymers, as well as its time dependence. As time passed, the stains of BSA faded away, which shows the progress of the reaction in both cases. The findings were in accordance with the OPA reaction, whereas a higher molar ratio and longer time are preferred for the conjugation. However, a significantly longer time (5 days) was required for the advanced reaction for LBG. This could be attributed to the branched structure, which may not be preferred for the reaction, similar to fucoidan [32].

All of these findings were supported by circular dichroism measurements. Electronic circular dichroism (ECD) spectroscopy is a well-established method in protein/peptide analysis [33]. It gives information about the secondary structure of the molecule and can be used for the estimation of the α-helical, β-folded, and disordered structural ratios in a molecule. If the measured samples cannot be compared directly because of different concentrations or other disrupting parameters, then the dissymmetry factor (g) or a dissymmetry spectrum may be an indicative tool to follow the changes [34]. Therefore, g-values were calculated and plotted for both conjugates. The progress of the reaction over time (3 h, 24 h, 48 h) could be well observed in the ALG CD spectra and g-values. Investigating the CD spectra of LBG conjugates, the results were in a strong correlation with previous measurements, namely, the 1:6 ratio for a 5-day treatment was better for the BSA conjugation. Our data were further supported by Kim et al., who described that 1 mol BSA could bind 2.5–7 mol galactomannan [8]. In this study, we presented that the 1:6 galactomannan ratio was more effective than the 1:3 ratio.

The solubility improvement of poorly water-soluble apigenin was investigated with the best obtained BSA–ALG 1:3 (48 h) and BSA–LBG 1:6 (5 days) conjugates. The detected Induced CD (ICD) spectra and the UV spectra revealed that the conjugates possess better solubility improving properties than the native BSA. This result is in accordance with the fluorescence spectroscopy measurements, where we could find a strong quenching mechanism, indicating the binding of apigenin to the Trp region of BSA. The measured ICD is also a versatile tool for the detection of protein–ligand interactions [35]. An achiral ligand with a good chromophore does not have a CD spectrum, but if a chiral surrounding perturbs the symmetry of the transition(s), then a new CD band will appear at the absorption wavelength range where the chromophore group absorbs. The appearance of new ICD band(s) serve(s) as clear evidence of the interaction between the host molecule and the ligand [36]: this could be observed for both conjugates. Interestingly, Liu et al. revealed that glycated human serum albumin has increased binding affinity to flavonoids, such as apigenin, which could affect the metabolism of these compounds in diabetic patients [37]. This finding can support our study, i.e., the binding affinity (therefore solubility) improving properties of modified albumin to apigenin could be valuable in drug delivery.

## 5. Conclusions

In this study, bovine serum albumin (BSA) bioconjugates were developed, and the production was optimized. Locust bean gum galactomannan (LBG) and alginate (ALG) with different molar ratios were used to synthesize albumin bioconjugates using the nonenzymatic Maillard reaction in order to evaluate their potential to increase the solubility of the poorly water-soluble flavonoid apigenin. The progress of the Maillard reaction and the characterization of the conjugates were demonstrated using several investigation methods: FTIR, DSC, fluorescence, and circular dichroism spectroscopy studies. The determination of the remaining free amino group with OPA reaction and sodium dodecyl sulfate–polyacrylamide gel electrophoresis (SDS–PAGE) confirmed the spectroscopic findings. Interestingly, to obtain protein bioconjugates with the Maillard reaction, the appropriate molar ratio and incubation time are necessary and mainly depend on the structure of the biopolymer. This present study showed that LBG needed at least a 1:6 molar ratio and a longer time for the reaction (5 days), but with ALG, the conjugation was completed within 48 h with a 1:3 molar ratio. The development of protein–polysaccharide conjugates could have a great advantage in terms of viscosity in drug delivery. Rheology has an importance in the human body since several body fluids, such as synovial fluid, tear film, or lung fluid, possess a viscoelastic behavior. Our data revealed that the rheological behavior of the bioconjugates was not altered: the LBG conjugates maintained the viscoelastic properties, which could play a role in drug delivery. The solubility-increasing properties of conjugates could take advantage in formulating BCS II drugs. All of these findings support the promising possibility of using albumin–biopolymer Maillard conjugates for drug delivery applications.

## Figures and Tables

**Table 1:** Experimental parameters of BSA conjugates.

Conjugates	Molar Ratio	Time
BSA-LBG	1:3 and 1:6	1, 3, 5 days
BSA-ALG	1:1, 1:3	3, 6, 8, 18, 24, 48 h

**Table 2:** Calculated viscosity values by linear interpolation process.

Sample	δ (°)	G’ (Pa)	G’’ (Pa)
BSA-LBG 1:3	43.7	42.01	40.14
	46.14	35.93	37.38
	45	38.77	38.66
BSA-LBG 1:6	48.2	2.866	3.206
	46.63	2.677	2.833
	45	2.48	2.45

## Data Availability

Not applicable.

## Supplementary Materials

The following are available online at https://www.mdpi.com/article/10.3390/biomedicines9070737/s1, Table S1: Process parameters of freeze drying.

## Abbreviations

ALG	Alginate
BSA	Bovine serum albumin
CD	Circular dichroism
ECD	Electronic circular dichroism
DSC	Differential scanning calorimetry
FTIR	Fourier transform infrared spectroscopy
HSA	Human serum albumin
ICD	Induced circular dichroism
IR	Infrared
LBG	Locust bean gum
MRP	Maillard reaction products
OPA assay	Ortho-phthaldialdehyde assay
PAS staining method	Periodic acid–Schiff staining method
SDS–PAGE	Sodium dodecyl sulfate–polyacrylamide gel electrophoresis
Trp	Tryptophan
