# Supplementary material for: Optimization and Development of Albumin–Biopolymer Bioconjugates with Solubility-Improving Properties
Source: Biomedicines. 2021 Jun 26;9(7):737. doi: 10.3390/biomedicines9070737 (PMC8301381; doi:10.3390/biomedicines9070737)
Supplement: Supplementary file 1 [file biomedicines-09-00737-s001.zip › biomedicines-1226974-supplementary.pdf]

Supplementary Materials

Table S1. Process parameters of freeze drying.

Time (Hours)	Temperature (°C)
0–4	−40
1	15
1	20
8	30
6	40
